# Blood Plasma Calorimetric Profiles of Women with Preeclampsia: Effect of Oxidative Stress

**DOI:** 10.3390/antiox12051032

**Published:** 2023-04-29

**Authors:** Regina Komsa-Penkova, Sashka Krumova, Ariana Langari, Ina Giosheva, Lidia Gartcheva, Avgustina Danailova, Lora Topalova, Tanya Stoyanova, Velichka Strijkova, Alexey Savov, Svetla Todinova

**Affiliations:** 1 Department of Biochemistry, Medical University—Pleven, Sv. Kliment Ohridski Str. 1, 5800 Pleven, Bulgaria; regina.komsa-penkova@mu-pleven.bg; 2 Institute of Biophysics and Biomedical Engineering, Bulgarian Academy of Sciences, “Acad. G. Bontchev” Str. 21, 1113 Sofia, Bulgaria; sashka.b.krumova@gmail.com (S.K.); arianalangari@abv.bg (A.L.); ina_gi@abv.bg (I.G.); avgustina_danailova@abv.bg (A.D.); topalovaloram@gmail.com (L.T.); tanya.sttoyanova@gmail.com (T.S.); vily_strij@abv.bg (V.S.); 3 National Genetics Laboratory, University Hospital of Obstetrics and Gynecology “Maichin Dom”, Medical University Sofia, Zdrave Str. 2, 1431 Sofia, Bulgaria; alexey.savov@abv.bg; 4 National Specialized Hospital for Active Treating of Haematological Diseases, 1756 Sofia, Bulgaria; l.garcheva@hematology.bg; 5 Institute of Optical Materials and Technologies “Acad. Yordan Malinovski”, Bulgarian Academy of Sciences, “Acad. G. Bontchev” Str. 109, 1113 Sofia, Bulgaria

**Keywords:** preeclampsia, blood plasma proteome, differential scanning calorimetry, oxidative stress, protein thermal stabilization, capillary electrophoresis, atomic force microscopy, protein aggregates

## Abstract

Preeclampsia is a pregnancy-related disease with poor placentation and presents itself through hypertension and proteinuria. The disease is also associated with the oxidative modification of proteins in maternal blood plasma. In this work, we combine differential scanning calorimetry (DSC), capillary electrophoresis, and atomic force microscopy (AFM) to evaluate the changes in the plasma denaturation profiles of patients with preeclampsia (PE) as compared with those of pregnant controls. Our results demonstrate that the last trimester of pregnancy substantially affects the main calorimetric characteristics of blood plasma from pregnant controls relative to nonpregnant women. These variations correlate well with the changes in protein levels determined by electrophoresis. DSC analysis revealed significant deviations in the plasma heat capacity profiles of preeclamptic patients from those of pregnant controls. These alterations are expressed mainly in a substantial reduction in albumin-assigned transitions and an upward shift in its denaturation temperature, lower calorimetric enthalpy changes, and a reduced ratio of heat capacity in the albumin/globulin-assigned thermal transitions, which are more pronounced in severe PE cases. The in vitro oxidation model shows that the alteration of PE thermograms is partly related to protein oxidation. AFM data detected numerous aggregate formations in the plasma of PE samples and fewer small ones in the pregnant controls, which are not found in healthy nonpregnant samples. These findings could serve as a basis for further investigations to reveal the possible relationship between albumin thermal stabilization, the increased inflammatory state and oxidative stress, and protein misfolding in preeclampsia.

## 1. Introduction

Preeclampsia (PE) is a severe pregnancy-related disorder characterized by new-onset hypertension and proteinuria, commonly occurring after 20 weeks of gestation [1]. It is one of the leading causes of fetal growth restriction (FGR) and maternal and neonatal mortality and morbidity. Despite many studies in this area, the etiology and pathogenesis of the disease are still not fully understood [2]. Compromised trophoblast invasion, vascular dysfunction, and maladaptation of the placenta are thought to play a central role in PE pathogenesis.

Preeclampsia is a multifactorial complication, and several subtypes are defined based on the time of the onset of clinical symptoms, their severity, the disease progression, and/or the presence of FGR.

PE is assumed to occur in two stages. The first changes are related to the impairment of the trophoblast invasion of the decidua. It has been suggested that failed decidual differentiation before pregnancy may contribute to impaired trophoblast invasion [3]. The shallow trophoblast invasion is related to the reduced proliferation of villous and extravillous cytotrophoblasts [4]. The failure of the extravillous trophoblast to sufficiently invade the uterine spiral arteries is supposed to be one of the factors in the occurrence of preeclampsia. Vascular dysfunction also plays a crucial role in the pathogenesis of PE. Potential abnormalities of vascular dysfunction include compromised placentation, impaired spiral artery remodeling, and endothelial damage, which are further intensified by an impaired balance of antiangiogenic and proangiogenic factors, immune molecules, and mitochondrial oxidative stress [5].

There is evidence that trophoblast immaturity results in placental hypoxia [6]. Defective spiral artery remodeling leads to reduced blood flow to the placenta and could be a contributing factor to placental lesions in PE [7]. Lesions can also be due to ischemia–reperfusion or hypoxia–reoxygenation-type damage caused by reactive oxygen species (ROS) [8].

Subsequent alterations in the expression of circulating soluble anti-angiogenic factors from the ischemic placenta or pro-inflammatory proteins precede the onset of the clinical signs involved in the pathogenesis of PE [9]. In response to inadequate blood flow to the placenta, it becomes extremely hypoxic and activates the release of soluble factors into the mother’s bloodstream. For example, the elevated serum level of soluble endoglin (sEng) and fms-like tyrosine kinase-1 (sFlt-1) correlate with the disease’s severity [10,11]. Recent studies have shown the presence of factors released by the injured endothelium in the circulation of women with PE, including endothelin-1, fibronectin, Von Willebrand factor, thrombomodulin, markers of oxidative stress, and inflammatory cytokines [9,12]. In preeclampsia, oxidative stress is found both in the placenta and in the maternal circulation blood flow. The initial placentation occurs in increased oxygen tension and a rise in the activity of several antioxidant enzymes. It is suggested that a reduction in the antioxidant response to the oxygenation stimulus, resulting in oxidative stress, may contribute to the impairment of trophoblast invasion [13]. Therefore, an impaired response to an oxidant stimulus could be one of the earliest events in preeclampsia.

Maternal serum from preeclamptic women shows evidence of the oxidative modification of proteins [14]. The circulating amyloid precursor transthyretin and amyloid precursor proteins were found to be misfolded in preeclampsia [15,16]. Several other proteins, such as alpha-1 antitrypsin, albumin, transthyretin, and IgG k-free light chains, appeared to be dysregulated in PE. Oxidized and aggregated forms of these proteins can be detected in the placenta, urine, and serum of PE patients. Human serum albumin (HSA) redox state changes are associated with various kinds of oxidative-stress-related diseases [17,18,19,20,21].

In recent years, there are a number of proposed predictive methods and serum markers of placental or vascular origin for PE occurrence, including uterine artery Doppler, mean arterial pressure, serum pregnancy-associated plasma protein-A (PAPP-A), placental growth factor (PIGF), fetal hemoglobin, Inhibin-A or sFlt-1, beta-human chorionic gonadotropin (β-hCG), and soluble endoglin (sENG). [22,23] In this regard, the detection of some factors for abnormal shallow trophoblastic invasions may be promising for the early detection of PE. Maternal deficiency in annexin A2 expression was found to influence aberrant decidualization and shallow cytotrophoblast invasions [24]. Fantone et al. demonstrated that AT-rich interactive domain 1A (known as BAF250a) appears to be a useful marker for poor trophoblast differentiation in PE and FGR [25].

However, the sensitivity and specificity of the proposed markers are not high enough, and they lack sufficient diagnostic accuracy to be used in daily routine practice. The prognosis and prevention of preeclampsia and gestational hypertension are still limited.

The application of new approaches for detecting markers in blood plasma/serum is of particular importance for non-invasive early diagnosis, which could allow for the initiation of PE treatment before its clinical manifestation. Over the past 10 years, differential scanning calorimetry (DSC) has been applied to the analysis of biofluids that are considered a rich source of potential diagnostic biomarkers [26,27,28,29,30,31,32,33,34]. Calorimetric profiles reflect the variations in the thermal stability of biofluids (such as blood, plasma/serum, cerebrospinal fluid, synovial fluids, and saliva) induced by alterations in the concentration and/or conformation of their protein constituents as a function of temperature.

We hypothesize that plasma proteins in preeclampsia are altered in concentration, structure, oxidation level, and/or binding interactions and that elucidating these alterations from a thermodynamic point of view will shed new insight into this disorder. For this purpose, in this work, we analyze the thermodynamic behavior of blood plasma derived from preeclamptic women and healthy normotensive pregnant controls (PC) in the third trimester of pregnancy. The results reveal, for the first time, specific calorimetric characteristics for PE plasma that, on the one hand, distinguish it from that of PC plasma and possess some similarities with the features of plasma subjected to oxidative stress on the other.

## 2. Results

### 2.1. Patients’ Characteristics

In this study, three groups of women were enrolled: (i) patients with preeclampsia (PE group), (ii) healthy normotensive women with normal pregnancies (PC group), and (iii) healthy nonpregnant women (NPC group). Table 1 reports the main participants’ characteristics, i.e., age, gestational week, gestational age at diagnostic and at delivery, the body weight of pregnant women, newborn weight, and the main biochemical characteristics (total plasma protein, albumin, and fibrinogen levels; platelet count; aspartate aminotransferase; and alanine aminotransferase). As can be seen, the biochemical parameters did not differ significantly between the PC and PE groups.

Of the presented cases, 22% had severe preeclampsia, requiring early delivery. According to gestational age at diagnosis, 42% of the studied PE cases were early onset. Of note, all included cases with severe PE were early onset, while among non-severe PE cases, only 30% were early onset. Preterm newborns occurred in 52% of the PE cases. The weight of neonates born to mothers with preeclampsia was 39% lower compared with those born to mothers with normal pregnancies (Table 1). According to the World Health Organization Fetal Growth Chart [35], 42.3% of newborns are below 10 percentiles, with 30.3% in non-severe cases and 82.3% in cases of severe PE.

### 2.2. DSC Blood Plasma Profiles of Healthy Nonpregnant Women and Pregnant Women in the Third Trimester of Pregnancy

**Figure 1:**
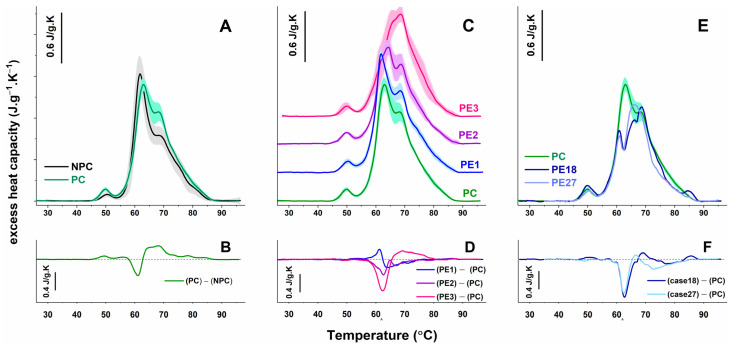
DSC profiles (average—solid lines; standard deviations—shading) of blood plasma from healthy nonpregnant women (NPC: black line, gray shadow) and normotensive pregnant women (PC: green line, cyan shadow) (**A**) and the studied groups of patients: PE1 (blue line/light blue shading), PE2 (violet line/violet shading), and PE3 (red line/pink shading) (**C**) and the ungrouped cases (PE18, dark blue line and PE27, light blue line) (**E**). For clarity, the thermograms in panel (**C**) are displaced vertically. The average thermogram registered for healthy pregnant normotensive women (PC, green line) in the third trimester of pregnancy is shown in panels (**C**,**E**). Difference plots represent the subtraction of the average NPC calorimetric curve from that of PC (panel (**B**)) and the subtraction of the average PC calorimetric curve from those of the PE scans (panels (**D**,**F**)). All thermograms are recorded with a scan rate of 1 °C·min^−1^ in a range of 27–95 °C.

The plasma thermograms recorded for healthy nonpregnant and pregnant women are presented in Figure 1. Similar to previously published reports by us and others [36,37], several thermal transitions can be observed in the calorimetric profiles of the blood plasma from healthy female subjects, which can be attributed to the denaturation of the most abundant proteins in plasma [38]. The main peaks in the low-temperature range (i.e., at 50 °C, 62 °C, and 68 °C) reflect the contribution of fibrinogen, albumin, and immunoglobulin denaturation, while the two shoulders (at 75 °C and 82 °C) in the high-temperature range (above 70 °C) originate mainly from the denaturation of complementary C3 proteins, IgA, IgG, transferrin, and IgG. It should be noted, as Garbett et al. [38] demonstrated, that other plasma proteins with a lower concentration also contribute to these transitions, albeit with less weight. An important parameter reflecting changes in major plasma proteins is the ratio of the maximum heat capacities of albumin (HSA) and immunoglobulin (Ig)-assigned transitions, c_P_^HSA^/c_P_^Igs^, which, for the healthy female controls in this study. was found to be 1.94 ± 0.3 (Table 2).

To clarify how the advancement of pregnancy affects the thermodynamic behavior of blood plasma proteome, we compared the thermograms of plasma derived from healthy pregnant women in the third trimester with those of nonpregnant female controls (Figure 1A). The PC thermograms differed mainly in the amplitude of the main transitions, i.e., fibrinogen (Fg) and Ig-assigned transitions had increased excess heat capacities. Although the mean value of c_P_^HSA^ decreased compared with the NPC thermograms (Table 2), no statistically significant difference was found. Another significant dissimilarity we found was a shift in the midpoint melting temperature of the albumin transition toward a higher temperature by 2 °C (Figure 1A, Table 2). As a result of these alterations, the weighted average center of the thermograms (T_FM_) and the enthalpy change (ΔH_cal_) were found to be higher than those determined for the NPC group (Table 2), which reveals the overall stabilization of the plasma proteome as compared with the nonpregnant controls.

### 2.3. DSC Plasma Profiles of Patients with Preeclampsia

To evaluate the influence of the PE pathology on the thermally induced denaturation of plasma proteins, the PE thermograms were compared with those of healthy control women carrying a singleton gestation at a similar gestational age (Figure 1C). The patients′ DSC profiles were classified into three groups according to the similarity in the shape of the thermograms, the c_P_^HSA^/c_P_^Igs^ ratio, and the midpoint melting temperature of the most abundant plasma protein, i.e., albumin. We also applied the statistical approach developed by Fish and coauthors for the analysis of PE thermograms [39].

It can be clearly seen that the calorimetric profiles of all three groups differ from that of PC, albeit to a different degree. The PE1 group, which includes only five of the studied cases, is the closest to the PC one. The T_FM_ and the total enthalpy were not significantly different from those of the control group (Table 2). The major transition, i.e., that of HSA, was destabilized by ca 1.2 °C and had a higher amplitude relative to the control one (Figure 1C). The average c_P_^ex^ value of the Ig-assigned transition was slightly lower compared with that of the PC group but lacked a statistically significant difference (*p* > 0.05). Consequently, the c_P_^HSA^/c_P_^Igs^ ratio was higher than the value of the PC set, and the similarity metric, ρ, determined for PE1 had a high value of 0.89 (Table 2).

The DSC profiles of the two other patient groups differed considerably from the PC set. For PE2 thermograms, the total enthalpy and the excess heat capacities of albumin and immunoglobulin transitions were significantly diminished. The albumin transition was also up-shifted by more than 1.5 °C compared with the corresponding transition in the PC group, and furthermore, a shoulder at about 61 °C appeared (Figure 1C). The shift to a higher-temperature HSA transition was even more pronounced in the PE3 group (more than 3 °C) with a simultaneous reduction in its c_P_^ex^ value. As a result, it almost overlapped with that of the Ig-assigned transition. The latter increased significantly in amplitude and thus became the dominant peak in the thermogram (Figure 1C). The reduced c_P_^HSA^/c_P_^Igs^ ratio, the only one that has a value less than one, and an increase in T_FM_ values were specific features of the PE3 thermograms (Table 2). Similar to the PE2 thermograms, a shoulder at 60 °C was also detected in the PE3 set. The combined similarity parameters, ρ, for the PE2 and PE3 groups were calculated to be 0.79 and 0.71, respectively (Table 2). The difference curve, representing the subtraction of the average DSC profile of the PC group from those of the PE groups, revealed the severity to which the PE thermograms differed from the control one (Figure 1D). The most prominent dissimilarity (both in intensity and in temperature range/interval) was found for the PE3 group. These difference plots were distinguished by a negative peak at about 62–63 °C due to the upshift in HSA denaturation for the PE2 and PE3 groups and a positive peak for the PE1 set, reflecting the opposite trend. At ca 68 °C, the positive peak for PE3 was enlarged, reflecting further deviation in the thermograms from the control one (Figure 1D).

Two of the patients’ thermograms, denoted PE18 and PE27, could not be classified into any of the defined groups (Figure 1E). A common feature of the thermograms of the third patient group was the strongly shifted HSA transition at higher temperatures, where, as a result of its overlap with the Ig peak, the latter became the dominant one. In these two thermograms, however, this phenomenon was even more pronounced, and the two transitions (i.e., that of HSA and Ig) overlapped. Furthermore, a well-distinguished sharp transition was established at 61 °C. In the case of PE18, the amplitude of the Fg transition was additionally elevated, and the last transition at about 86 °C was clearly defined. The difference curve (Figure 1D) revealed the extended range in which the two individual DSC curves differed significantly from that of the PC one.

It should be noted that PE27 and four of the cases (44%) of the PE3 group have severe preeclampsia vs. one case (9%) in the PE2 set.

### 2.4. DSC Blood Plasma Profiles of Patients with Severe vs. Non-Severe Preeclampsia

**Figure 2:**
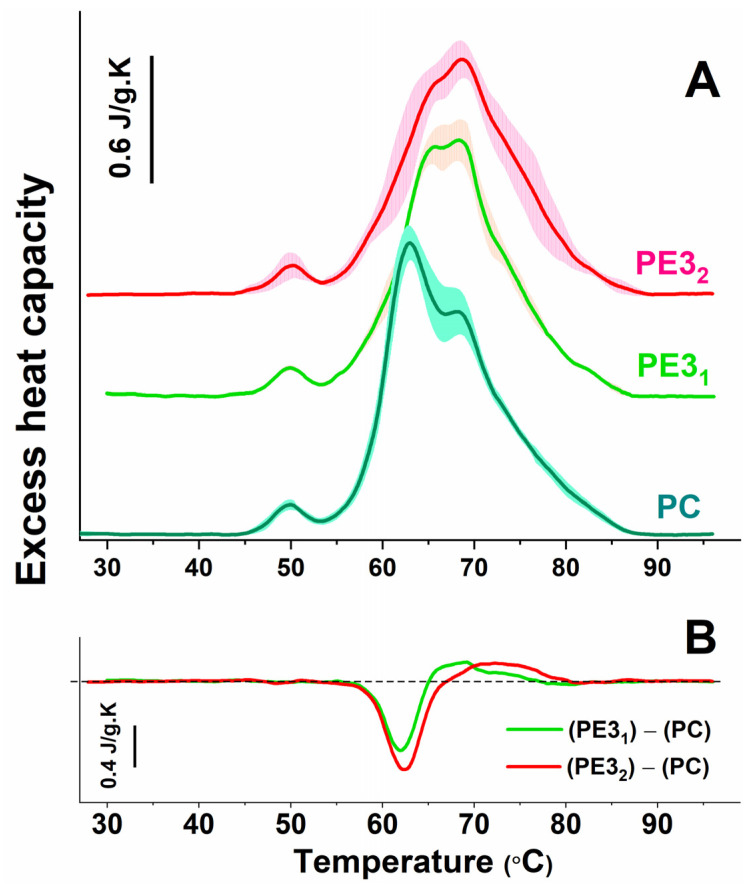
DSC profiles (average—solid lines; standard deviations—shading) of blood plasma from control pregnant women (PC: green line, cyan shadow) and patients with non-severe (PE3_1_: light green line, orange shadow), and severe (PE3_2_: light green line, orange shadow) PE (**A**). The difference plots for the subtraction of the average PC calorimetric curve from those of the PE3_1_ and PE3_2_ scans are presented in panels (**B**).

In order to elucidate to what extent the severity of the disease affects the deviation of the PE thermograms from the control ones, we further divided the PE3 group into two subsets, the first (PE3_1_) comprising blood plasma thermograms from patients with non-severe preeclampsia and the second one (PE3_2_) from those with severe preeclampsia (Figure 2A). It can be clearly seen that the main calorimetric characteristics of the PE3_2_ subgroup differed to a greater extent from the controls and from those in the PE3_1_ subset (Table 3). The c_P_^HSA^/c_P_^Igs^ ratio of PE3_2_ was 10% lower than that of PE3_1_. A lower enthalpy change (ΔH_32_) was found in PE3_2_, while the T_FM_ values were further increased by approximately 1.2 °C compared with the PE3_1_ thermograms, with more than 2 °C vs. the controls (Table 3). The amplitude of the peak at about 63 °C of the difference curve was larger as compared with that of PE3_1_, and the extended range above 67 °C revealed a more substantial difference in PE3_2_ in comparison with the controls (Figure 2B).

### 2.5. Protein Fractions of PC and PE Samples

Table 4 summarizes the results obtained via capillary electrophoresis. The data analysis revealed that all protein fractions determined for the PC group differed substantially from those of the NPC group. The albumin value was significantly decreased as compared with the NPC group. A two-fold increased level of α1- (comprising α1-antitrypsin, thyroid-binding globulin, and transcortin), α2- (which consists mainly of ceruloplasmin, α2-macroglobulin, and haptoglobin), and β2-globulin (composed mainly of complementary C3 and IgA proteins; fibrinogen also migrates in the β2-fraction) fractions was established (Table 4). The Β1-fraction (mainly containing transferrin) was also above the value of the NPC group, while γ-globulin fractions differed slightly and had lower values.

As noted above, the statistical analysis for the patients’ data was performed by comparing them with the PC data. For the PE1 and PE3_2_ groups, no difference was found for any of the fractions compared with the PC values. For the PE2 group and PE3_1_ subset, only α2-and γ-globulins, respectively, were found to be lower compared with those of the PC values, while the remaining bands were within the reference limits.

The two ungrouped cases (PE18 and PE27) differed in some of the electrophoretic bands from the control ones. For PE18, the albumin level was significantly reduced, while the two α-fractions were almost twice as high compared with the values of the pregnant controls. An elevated level of the γ-globulin fraction compared with the PC group was also detected. Contrary to the results from the PC group and the PE18 case, a higher γ-fraction and lower β1-fraction were determined for PE27. The remaining fractions did not differ from the control ones.

### 2.6. Oxidative Stress Simulation

**Figure 3:**
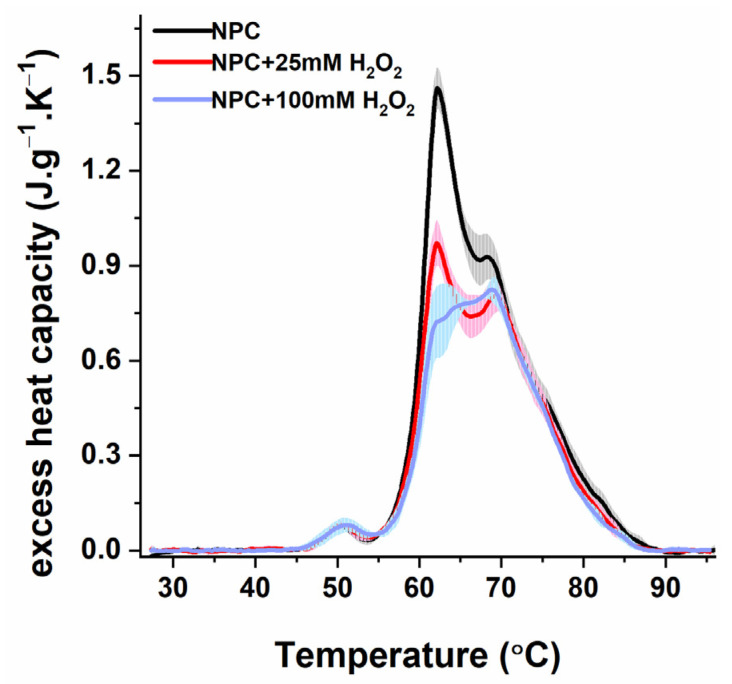
Average DSC profiles of blood plasma from healthy nonpregnant women (NPC: black line, gray shadow) and NPC plasma treated with 25 mM (red line, pink shadow) and 100 mM of H_2_O_2_ (blue line, light blue shadow).

Oxidative stress has been suggested to be one of the factors in the pathophysiology of preeclampsia. To elucidate to what extent oxidative stress affects the thermodynamic behavior of blood plasma, and HSA in particular, in PE, we mimicked oxidative stress in newly isolated plasma from healthy females using a treatment with H_2_O_2_, a physiological oxidant produced in the body and widely used in model systems. The treatment of plasma with 25 mM H_2_O_2_ led to a strong reduction in the HSA-assigned peak (Figure 3), resulting in a lower c_P_^HSA^/c_P_^Igs^ ratio and a calorimetric enthalpy change (Table 5). However, the overall shape of the heat capacity curve did not change significantly compared with the control one, and no shift in the main transition was observed.

The exposure of plasma to 100 mM of H_2_O_2_ further decreased the HSA peak and enthalpy value (Figure 3). Moreover, the calorimetric profile differed significantly from the average non-treated plasma curves. Although the thermograms of the oxidized plasma did not entirely resemble those of the PE sets, some features in common with the PE2 and PE3 groups can be found, such as the split of the HSA transition, the substantial reduction in its amplitude, and the c_P_^HSA^/c_P_^Igs^ ratio. Similar to the thermograms of PE3 and the two ungrouped cases, the Ig transition became the dominant one in the 100 mM oxidized plasma thermograms.

**Figure 4:**
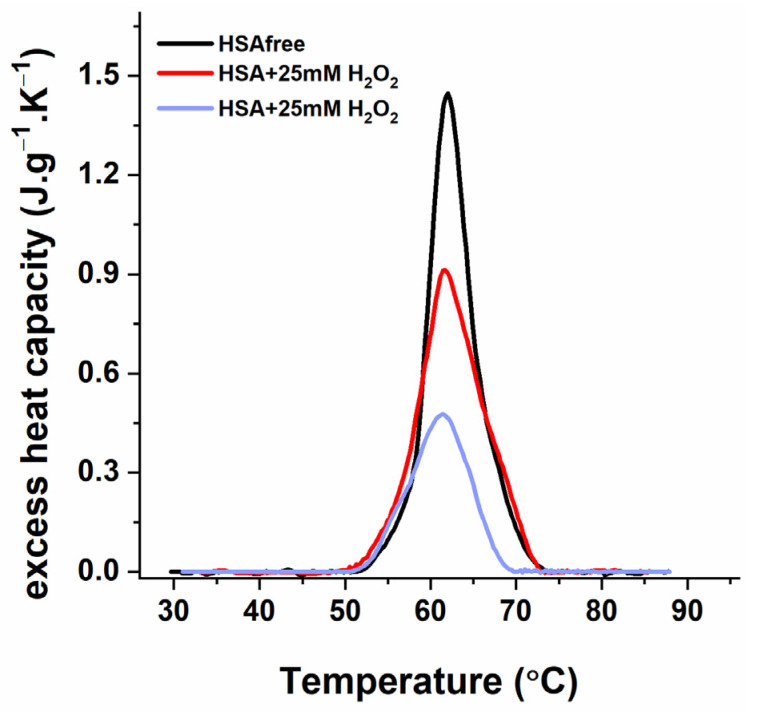
DSC profiles of purified HSA treated with 25 mM and 100 mM of H_2_O_2_, as indicated in the figure legend. Scan rate, 1 °C·min^−1^.

The treatment of isolated albumin (fatty-acid-free) with the same concentrations of hydrogen peroxide also led to a substantial decrease in the heat capacity and enthalpy of the DSC profile but without a shift in its midpoint denaturation temperature (Figure 4, Table 6).

### 2.7. Atomic Force Microscopy Analysis of Plasma

**Figure 5:**
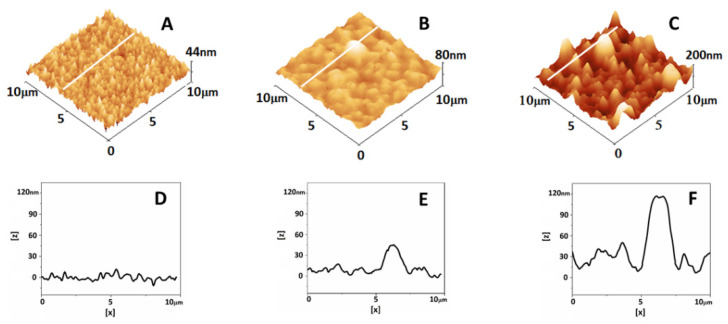
Representative 3D AFM images of blood plasma derived from healthy nonpregnant women (NPC) (**A**), pregnant women (PC) (**B**), and patients with preeclampsia (PE) (**C**) and cross-section plots (**D**–**F**) corresponding to the white lines in (**A**–**C**). The images were taken in contact mode in air at room temperature.

An increased inflammatory state, associated with pregnancy and particularly with pregnancy complications, is a prerequisite for protein misfolding, which can result in aggregate formations. To check this probability, we analyzed the plasma of women in the studied groups using AFM. As shown in Figure 5A, the plasma proteins of the NPC group appeared to be homogenously distributed over the glass surface. Small aggregates (with a height of 35 ± 7 nm and a diameter of 1.6 ± 0.4 µm) with approximately spherical shapes were found in the PC samples (Figure 5B). The AFM analysis revealed larger (height, 79 ± 26 nm; diameter, 2.4 ± 0.8 µm) and more numerous aggregates for the PE samples (Figure 5C). Different roughness values (R_rms_) calculated on scanned areas of 1 × 1 µm^2^, free of aggregates (for the PC and PE samples), were found for the NPC, PC, and PE groups. The R_rms_ of the PC group were twice as high compared with those of the NPC group (7.6 ± 0.9 nm vs. 3.6 ± 0.3 nm, respectively). The highest roughness value was determined for the PE samples (18.9 ± 3.9 nm).

## 3. Discussion

### 3.1. Changes in Blood Plasma Proteome in the Last Trimester of Pregnancy

In recent years, DSC has emerged as a new approach to distinguish various diseases from a healthy status based on the analysis of the thermodynamic features of blood plasma proteome. In line with these efforts, this study demonstrates, for the first time, specific characteristics of the plasma denaturation profiles of patients with preeclampsia that distinguish them from those of healthy pregnant women in the third trimester of pregnancy.

Well-defined reference thermograms for a specific condition, such as pregnancy, are essential for the analysis of pathological conditions. Our recent study showed that early pregnancy did not induce significant changes in the thermodynamic behavior of plasma proteins [37]. Herein, however, we showed that late pregnancy significantly alters plasma calorimetric profiles. The increase in the enthalpy change, ΔH_cal_, and weighted average center, T_FM_, of thermograms from healthy pregnant women in the third trimester of pregnancy compared with that of nonpregnant women might be related to an increase in the concentration of some plasma proteins or/and protein stabilization. Indeed, the correlation with the electrophoretic data revealed that changes in the calorimetric characteristics are, to a great extent, due to changes in protein concentrations. The established alterations in the amplitudes of the observed peaks in the PC thermograms showed a high positive relationship with the corresponding changes in the levels of the protein fractions determined via electrophoresis analysis. The regression analysis revealed a high correlation between the amplitude of the Ig transition and the levels of α-globulin (r = 0.77) and β2-globulin (r = 0.84) fractions. It has been found that the concentrations of α- and β-globulins in uncomplicated pregnancies are raised compared with the nonpregnant state, with the highest percentage increase for α-1-globulin [40]. The elevated level of estrogen is suggested to be involved in the changes in plasma protein concentrations [41]. The nearly 1.5-fold increase in plasma volume during pregnancy has been assumed to be another factor in the altered protein concentrations in pregnancy [42]. In line with this, the drop in the HSA peak we found in the PC thermograms most probably reflects the lower albumin level in the blood serum in pregnant controls. However, the well-distinguished upshift in the albumin-assigned transition could not be related to the proteins’ level alterations but rather to the HSA redox state and binding with pregnancy-specific molecules or proinflammatory factors.

Pregnancy is also associated with an increased fibrinogen concentration, which is part of the adaptation of the coagulation system during gestational progress [43]. As expected, the enlarged Fg transition of the PC calorimetric curves strongly correlates (r = 0.91) with the elevated level of fibrinogen determined via hematological analysis.

### 3.2. Deviations in Calorimetric Profiles and Blood Plasma Proteome in Preeclampsia

As a multifactorial complication, preeclampsia is commonly classified into several subtypes, defined based on the time of onset, as early and late preeclampsia; the severity of the symptoms and progression, as severe and non-severe; and/or the presence of FGR. In this study, the patients were first classified according to similarities in their thermal profiles into the PE1, PE2, and PE3 groups, and those groups were further explored for correlation with the time of onset (early and late PE) and the severity of the disease. This approach revealed that the PE1 group’s main characteristics were very close to the pregnant controls, except for deviation in the albumin denaturation temperature. This group included only non-severe cases with a higher percentage of late-onset preeclampsia. It is important to note that almost all thermodynamic characteristics were in a range between the NPC and PC groups, perhaps as a consequence of their mean GA being 34 weeks, as it is less than that of the PC group (37 weeks).

A more substantial difference in the calorimetric characteristics (a significant change in the thermal stability of albumin and a change in total enthalpy) was observed in the PE2 group. This group had an almost equal distribution of early- and late-onset preeclampsia (45.5% vs. 54.5%) and only one severe case.

A strong deviation in the PE3 thermograms compared with those of the PC group was observed. Almost all severe cases were in this group, and all of them were early onset. The thermodynamic characteristics were significantly altered when compared with the PC group. This may be associated with differences in the levels of the plasma components, the interaction of the main plasma constituents with specific pathological molecules, or other pathological-associated factors.

In our investigated group of patients, 22% were severe cases, all of which were early onset and distributed in the PE3 group (except for one case in the PE2 group and case PE27).

A data analysis of the most altered thermograms, i.e., the two subsets of the PE3 group, including non-severe (PE3_1_) and severe (PE3_2_) cases, confirmed the influence of disease severity on the extent of change in the thermodynamic features of the patients’ plasma proteomes. The applied statistical analysis supports the relationship between the PE severity and the trend in worsening thermogram parameters, i.e., the stronger suppression of the albumin-assigned transition, the more substantial the decrease in the enthalpy change, resulting in the most upward shift in the weighted average center. However, to clarify and confirm this relationship, more cases of severe PE should be studied.

As no difference was detected in the protein fractions between the PE groups and the control one, the altered calorimetric shape expressed mainly within the lowering and accompanying shift in the HSA transition could be due to factors other than the protein concentration. It is known that under certain pathophysiological conditions, albumin can undergo conformational changes upon ligand binding [44], which, in turn, can affect its thermodynamic behavior. Hence, it can be assumed that a fraction of the HSA is stabilized as a consequence of its binding with some PE-specific proteins. Several studies report that, in preeclampsia, some placenta-related markers, such as leptin, Inhibin-A, or sFlt-1, differ in concentration compared with uncomplicated pregnancies [45,46]. Significantly raised activin A, pregnancy-associated plasma protein A (PAPP-A), and sE-selectin were found more often in women with preeclampsia compared with the controls. Bersinger et al. suggest that activin A could serve as the better predictive serum marker for this pathology due to its greatest increase compared with the other PE markers [47]. Therefore, it would be interesting to study if these proteins interact with albumin.

The samples of the two ungrouped cases showed almost identical denaturation curves but strongly differed in their electrophoretic profiles. Therefore, the apparent similarity between their DSC profiles on the one hand and the strong alteration of their thermodynamic characteristics compared with the control and other PE groups on the other could not be explained solely by the protein level variations determined via electrophoresis. The common specific features of these cases were the clearly defined sharp transition at 60 °C, which could be due to the even more pronounced HSA stabilization, particularly in severe PE cases, thus shifting toward higher temperatures that expose an otherwise hidden transition at 60 °C. A unique characteristic of the PE18 case was the enlarged IgG/transferrin-assigned transition at 85 °C and the increased amplitude of the fibrinogen peak. Unexpectedly, the globulin fractions, i.e., of β1-and β2-globulins, where these proteins migrate did not differ from those of the control one. Therefore, the change in the calorimetric transitions of Fg and IgG/transferrin is likely due to their altered conformational state. All of the above-mentioned observations support our suggestion that the observed changes in the thermodynamic behavior of the plasma proteins in PE samples may be related to other pathological factors.

### 3.3. Simulation Experiments on the Effect of Oxidative Stress

In this work, we also evaluated if the previously stated alterations are related to oxidative stress. Albumin is the dominant antioxidant in plasma and can undergo structural modifications in response to oxidative stress [48]. This would inevitably alter the plasma DSC profile. It is well known that pregnancy leads to increased oxidative stress, mainly produced by a normal systemic inflammatory response, which results in high amounts of circulating reactive oxygen species (ROS) and reactive nitrogen species (RNS). The imbalance between the antioxidant mechanism and ROS/RNS can have a tremendous effect on pathological processes, e.g., preeclampsia [49].

Simulation experiments on the effect of oxidative stress induced by hydrogen peroxide demonstrate a dose-dependent alteration of the plasma thermograms compared with healthy females. Although the in vitro oxidation model does not result in transformations identical to those found in PE thermograms, some correlations can be made. The DSC experiments with the low-concentration (25 mM) H_2_O_2_ treatment indicate a strong reduction in the albumin and Ig-assigned transitions and a decrease in their enthalpy change. Furthermore, the plasma treatment with a four-fold-increased H_2_O_2_ concentration (100 mM) results in a significant shape transformation in the calorimetric curve. The thermograms of samples treated with 100 mM of H_2_O_2_ demonstrated some characteristics in common with those of the PE DSC profiles, i.e., the split and upshift of the HSA peak and increase in the Ig transition. The exposure of the purified HSA to the H_2_O_2_ alters the enthalpy change but not the protein denaturation temperatures. It should be noted that a drastic increase in the H_2_O_2_ concentration resulted in a much weaker decrease in the heat capacity of the plasma HSA peak compared with that of the purified albumin, suggesting the influence of the unimpaired antioxidant activity of the controls.

The results of H_2_O_2_ exposure suggest that the excessive oxidative stress on the one hand and the weakening of the protective machinery against ROS on the other could be partially responsible for the significant alteration found in the plasma proteomes of preeclamptic women. The decreased activity of glutathione peroxidase during preeclampsia has been reported to contribute to increased oxidative stress levels. Therefore, preeclampsia is characterized by an imbalance between the endogenous antioxidant system and free radicals, mostly ROS [50].

The thermodynamic behavior of PE blood plasma can also be affected by the presence of misfolded proteins, which are known to be one of the characteristics of preeclampsia. The AFM analysis revealed numerous aggregates in the plasma of PE samples and small ones in the PC that were not found in healthy nonpregnant subjects. Increased inflammatory stress in pregnancy is a condition that prompts protein misfolding in the mother’s organism. It is not yet fully understood how the maternal organism deals with protein misfolding in an uncomplicated pregnancy. However, it has been suggested that pregnancy-zone protein efficiently inhibits the aggregation of misfolded proteins [19]. The overexpressed ROS/RNS in preeclampsia may lead to the dysregulation of many proteins and consequently result in the toxic deposition of aggregates in body fluids [21,51]. High levels of aggregates of misfolded proteins have been observed in PE placental samples [52]. Further detailed research in this direction is needed in order to reveal plausible relationships between albumin thermal stabilization, the level of oxidative stress, and the presence of misfolded proteins in PE and elucidate and confirm the relationship between the severity of PE and changes in the specific calorimetric features of plasma proteins.

#### Limitations

The major limitations of this study were the low number of cases with severe preeclampsia vs. non-severe ones. Despite these limitations, our study is the first of its kind to investigate the influence of the disease on the thermodynamic behavior of plasma proteome alteration. To determine the DSC’s ability to discriminate disease severity, a future study with an increased number of patient samples representing different degrees of PE complications is needed. We believe that large-scale studies will be useful in clarifying the modulating factors of these modifications.

## 4. Patients and Methods

### 4.1. Study Groups and Ethics Statement

Twenty-seven patients (mean age, 26.9 ± 3.2 years; PE) diagnosed with PE admitted to the Medical University—Pleven and the Hospital of Obstetrics and Gynecology “Maichin Dom”, Medical University Sofia, between February 2019 and February 2023 were recruited in this study. The diagnosis of PE was defined according to hypertension in pregnancy guidelines [53]. Fourteen other age-matched (mean age, 27.3 ± 3.4 years; PC) normotensive pregnant women who developed neither PE nor other complications were selected as controls. Their gestational age was comparable to that of the preeclamptic women. Nineteen healthy, age-matched nonpregnant women (mean age, 29.8 ± 5.5 years; NPC) were also evaluated and served as controls. The study included only single pregnancies. Patients with multiple pregnancies, genetic disorders, uterine anatomical abnormalities, hormonal abnormalities (thyroid), concomitant infectious causes, antiphospholipid syndrome, immune disorders, and metabolic disorders such as diabetes were excluded from the investigation.

All subjects included provided written informed consent for the investigation. The study was approved by the Ethics Committee of Medical University—Pleven (approval No. 404-KENID 22/10/15) and was performed in accordance with the Helsinki International Ethical Standards on Human Experimentation.

### 4.2. Blood Collection

For the PE group, blood samples were taken at the time of diagnosis before medication administration. Blood samples (12 mL) were collected via intravenous puncture into two tubes (Vacutainer; Becton Dickinson, and Company, Franklin Lakes, NJ, USA) containing K3EDTA. The blood from the first vacutainer was used for DNA analysis, and the one from the second was used for plasma isolation.

### 4.3. Sample Preparation

Blood plasma was obtained after blood centrifugation at 1200× *g* for 15 min at 4 °C. The yellowish supernatant was separated and transferred to a new tube and further diluted in PBS buffer (140 mM NaCl, 2.7 mM KCl, 8 mM Na_2_HPO_4_, 1 mM KH_2_PO_4_) to the required concentration for DSC measurements.

### 4.4. Protein Content Analysis

The total protein content was determined via the Biuret method [54]. Capillary electrophoresis (Capillarys 2, Sebia, Lisses, France) was carried out to determine the levels of the main plasma protein fractions (HSA and α-, β-, and γ-globulins).

### 4.5. Plasma and HSA Oxidation

#### 4.5.1. Plasma Oxidation

Blood plasma from 5 healthy nonpregnant donors was isolated using the procedure described in Section 4.3. In total, 150 µL of plasma was diluted with 650 µL of PBS buffer (140 mM NaCl, 2.7 mM KCl, 8 mM Na_2_HPO_4_, 1 mM KH_2_PO_4_) and treated with two contestations (25 mM and 100 mM) of freshly prepared hydrogen peroxide (H1009 Sigma Aldrich, Pty Ltd., an affiliate of Merck KGaA, Darmstadt, Germany) from a stock solution of 1 M H_2_O_2_. The samples were incubated for 4 h at 4 °C. The oxidation reaction was stopped by 100 µL of 10 mM EDTA. Any excess oxidants were removed via intensive dialysis against a PBS buffer.

#### 4.5.2. HSA Oxidation

HSA-fatty-acid-free product (Sigma Aldrich) was used for oxidation. This was performed according to a standard protocol involving the incubation of HSA solution (2 mg/mL) in PBS buffer with two concentrations (25 mM and 100 mM) of freshly prepared hydrogen peroxide (H1009 Sigma Aldrich) from a stock solution of 1 M H_2_O_2_. The oxidation reaction was stopped using 100 µL of 10 mM EDTA. Any excess oxidants were removed via intensive dialysis against a PBS buffer.

### 4.6. DSC Experiments

DSC measurements were performed with a microcalorimetric system DASM-4 (Biopribor, Pushchino, Russia) with a cell volume of 0.47 mL. The samples were heated at a 60 °C/h scanning rate from 30 °C to 95 °C and preceded by a baseline run with buffer-filled cells. A constant pressure of 2 atm was applied to the cells to prevent any degassing of the solutions. The reversibility of the thermal transition was estimated by checking the reproducibility of the calorimetric trace during a second heating. Calorimetric curves were obtained after subtracting the second scans from the corresponding data of the first ones and then normalized to the plasma protein concentration. The calorimetric data were analyzed using the Origin Pro 2018 software package. The following calorimetric parameters were used: transition temperature (T_m_) and excess heat capacity (c_P_^ex^) of the successive thermal transitions; the ratio of the heat capacities corresponding to the most abundant plasma proteins, c_P_^HSA^/c_P_^Igs^; the total calorimetric enthalpy (ΔH_cal_) of the DSC profile (calculated as the integrated area under the heat capacity curve); and the weighted average center of the thermograms (T_FM_).

### 4.7. AFM Measurements

AFM imaging was performed on an Atomic Force Microscope (MFP-3D, Asylum Research, Oxford Instruments, Santa Barbara, CA 93117, USA). All AFM measurements were performed in contact mode at room temperature. Silicon AFM tips (Nanosensors, type qp-Bio) with a 50 kHz resonance frequency, 0.3 N/m of nominal spring constant, and a nominal tip radius of 8 nm (the shape is conical) were used.

For AFM imaging experiments, 200 µL of plasma was deposited on a clean coverslip and incubated for one hour at 37 °C to ensure maximal adsorption for each sample.

Morphology observation and morphometric characterization (height and diameter of aggregates in the PC and PE samples) were achieved using the Gwyddion and IgorPro 6.37 software. The roughness analysis was performed in scanning areas of 1.0 × 1.0 µm^2^, free of aggregates. The R_rms_ value was calculated as the mean square root of the height distribution as follows:

(1) $$R_{\mathrm{rms}}=\sqrt{\sum_{i=1}^{N}\frac{{\left(z_{i}-z_{m}\right)}^{2}}{\left(N-1\right)}}$$

where *N* is the total number of points, *z_i_* is the height of the *i*th point, and *z_m_* is the mean height.

### 4.8. Statistical Analysis

Data were reported as mean ± standard deviation (SD). Statistical significance was accepted at *p* < 0.05 for all comparisons. The nonparametric Mann–Whitney U test for independent samples was used to assess the statistical significance of differences between means. The linear relationship between the calorimetric characteristics of plasma samples, the levels of electrophoresis, and laboratory data were assessed using Pearson correlation analysis. The statistical methodology developed by Fish et al. [39] was applied to determine the degree of deviation/similarity between a set of reference thermograms (in this work, the PC set) and test thermograms using a similarity metric parameter, ρ, which combines two factors: similarities in shape (Pearson’s correlation coefficient, r) and in space (spatial distance metric, P).

## 5. Conclusions

For the first time, the denaturation profiles of plasma proteins from patients with preeclampsia were examined in relation to those of healthy pregnant normotensive women in the third trimester of pregnancy.

The obtained results demonstrate that late pregnancy substantially affects the main calorimetric characteristics of blood plasma from pregnant controls as compared with those of nonpregnant women. These variations correlate well with changes in protein levels determined via electrophoresis.

DSC analysis revealed significant alterations in the plasma heat capacity profiles of preeclamptic patients compared with those of the pregnant controls, manifesting in a significant reduction in the albumin-assigned transition and an upward shift in its denaturation temperature, lower enthalpy changes in the PE thermograms, and a reduced ratio of the heat capacity of the albumin/globulin-assigned thermal transitions. Furthermore, the severe PE cases were characterized by even significant deviations in the thermograms compared with the controls. The in vitro oxidation model shows that the alteration in PE thermograms is partly related to protein oxidation.

Atomic force microscopy data detected numerous large aggregates in the plasma of PE samples and fewer small ones in the pregnant controls but not in healthy nonpregnant samples. These findings could serve as a basis for further investigation to reveal the possible relationship between albumin thermal stabilization, the increased inflammatory state and oxidative stress, and protein misfolding in PE.

## Figures and Tables

**Table 1:** Patient characteristics (maternal age (years), mean blood pressure (BP), gestational week (GW), gestational age (GA) at diagnostic and at delivery, body weight (BW), and newborn weight) and biochemical parameters (total protein (TP), human serum albumin (HSA), fibrinogen (Fg) level, C-reactive protein (CRP), platelet count, aspartate aminotransferase (ASAT), and alanine aminotransferase (ALAT)), determined for nonpregnant (NPC) and pregnant controls (PC) in the third trimester of pregnancy and patients with preeclampsia (PE).

Characteristic	Reference Values for Pregnant Women	NPC (N = 19)	PC (N = 14)	PE (N = 27)
Maternal age (years)		29.8 ± 5.5	27.3 ± 3.4	31.2 ± 4.5
Mean BP (systolic/diastolic)		110 ± 7/74 ± 5	104 ± 6/72 ± 3	155 ± 11/104 ± 7
Gestational week (interval)		-	27–38	27–36
GA at diagnostic of PE		-	-	29.3 ± 3.2
GA at delivery		-	39.0 ± 1.09	33.9 ± 4.6
Body weight (kg)		57 ± 8	87 ± 7	92 ± 5
BMI (kg/m^2^)		21.4 ± 1.5	31.1 ± 1.8	32.8 ± 1.6
Newborn weight (g)		-	3376 ± 349	2144 ± 999
Proteinuria (mg in 24-h urine collection)		-	-	1090 ± 0.5
TP (g/L)	63–84	71.45 ± 3.11	68.34 ± 3.52	65.36 ± 7.42
HAS (g/L)	35–50	47.1 ± 1.77	39.54 ± 3.68	37.60 ± 4.79
Fg (g/L)	2.90–6.50	3.1 ± 0.56	5.32 ± 0.58	5.27 ± 1.22
CRP (mg/L) (interval)	0.5–5.0	0.37–1.12	5.1–19.9	0.93–95.35
Platelet Count × 10^9^/L	146–429	289 ± 73	254 ± 60	236 ± 99
ASAT (U/L)	4–32	21.1 ± 2.7	23.2 ± 3.4	19.9 ± 7.3
ALAT (U/L)	3–30	18.1 ± 3.0	14.9 ± 1.5	16.9 ± 8.3

Note: one patient diagnosed at 24 gestational weeks was also included in the study.

**Table 2:** Thermodynamic parameters (mean value ± SD) estimated from the calorimetric profiles of blood plasma from healthy nonpregnant (NPC) and pregnant controls (PC) and patients with preeclampsia (PE1, PE2, and PE3 groups): excess heat capacity (c_P_^ex^); transition temperature (T_m_) of the successive calorimetric transitions; the ratio of the specific heat capacities of HSA and Ig-assigned transitions (c_P_^HSA^/c_P_^Igs^); enthalpy change (∆H_cal_); the weighted average center (T_FM_) of the thermograms; and statistical metrics r, P, and ρ (according to Fish et al., 2010 [39]).

Groups	c_P_^Fg^ (J·g^−1^·K^−1^)	T_m_^HSA^ (°C)	c_P_^HSA^ (J·g^−1^·K^−1^)	c_P_^Igs^ (J·g^−1^·K^−1^)	c_P_^HSA^/c_P_^Igs^	∆H_cal_ (J·g^−1^)	T_FM_ (°C)	P	r	ρ
NPC (19)	0.08 ± 0.016	61.5 ± 0.6	1.54 ± 0.17	0.79 ± 0.1	1.94 ± 0.2	17.6 ± 0.5	65.2 ± 0.4			
PC (14)	0.15 ± 0.017 *	63.0 ± 0.3 *	1.39 ± 0.07	1.10 ± 0.11 *	1.29 ± 0.16 *	19.7 ± 0.8 *	66.4 ± 0.3 *	-	-	-
PE1 (5)	0.15 ± 0.01	61.6 ± 0.3 **	1.44 ± 0.13	0.97 ± 0.1	1.49 ± 0.17	19.0 ± 0.24	65.9 ± 0.4	0.92 ± 0.04	0.86 ± 0.03	0.89 ± 0.03
PE2 (11)	0.14 ± 0.02	64.2 ± 0.2 **	1.21 ± 0.12 **	1.0 ± 0.11	1.22 ± 0.07	18.0 ± 0.17 **	66.8 ± 0.2	0.76 ± 0.04	0.88 ± 0.04	0.79 ± 0.04
PE3 (9)	0.14 ± 0.03	66.3 ± 0.2 **	1.14 ± 0.14 **	1.2 ± 0.13	0.95 ± 0.06 **	18.0 ± 0.18 **	67.5 ± 0.3 **	0.51 ± 0.03	0.80 ± 0.02	0.71 ± 0.02
Case18	0.18 **	65.9	0.95	1.1	0.86	16.9 **	67.4 **	0.58	0.76	0.71
Case27	0.13	-	-	1.16	-	15.6 **	66.7	0.60	0.75	0.70

* Indicates statistically significant difference (*p* < 0.05) in the PC value compared with the NPC values. ** Indicates statistically significant difference (*p* < 0.05) in the PC control values.

**Table 3:** Thermodynamic parameters (mean value ± SD) estimated from the calorimetric profiles of blood plasma from control pregnant women (PC) and patients of the two subsets (PE3_1_, non-severe; PE3_2_, severe preeclampsia): excess heat capacity (c_P_^ex^); transition temperature (T_m_) of the successive calorimetric transitions; the ratio of the specific heat capacities of HSA and Ig-assigned transitions (c_P_^HSA^/c_P_^Igs^); enthalpy change (∆H_cal_); weighted average center (T_FM_) of the thermograms; and statistical metrics r, P, and ρ (according to Fish et al., 2010 [39]).

Groups	T_m_^HSA^ (°C)	c_P_^HSA^ (J·g^−1^·K^−1^)	c_P_^Igs^ (J·g^−1^·K^−1^)	c_P_^HSA^/c_P_^Igs^	∆H_cal_ (J·g^−1^)	T_FM_ (°C)	P	r	ρ
PC (14)	63.0 ± 0.3	1.39 ± 0.07	1.10 ± 0.11	1.29 ± 0.16	19.7 ± 0.8	66.4 ± 0.3			
PE3_1_ (5)	65.6 ± 0.2 *	1.19 ± 0.08	1.23 ± 0.091 *	0.96 ± 0.05 *	18.0 ± 0.19 *	67.2 ± 0.2 *	0.55 ± 0.05	0.81 ± 0.02	0.73 ± 0.02
PE3_2_ (4)	65.6 ± 0.2 *	0.98 ± 0.07	1.14 ± 0.06 *	0.86 ± 0.06 *	17.5 ± 0.14 *	68.4 ± 0.4 *	0.49 ± 0.03	0.78 ± 0.02	0.69 ± 0.02

* Indicates statistically significant difference (*p* < 0.05) in the PC control values.

**Table 4:** Concentration of the main plasma protein fractions (mean ± SD, presented as a percentage of the total protein content) determined via capillary electrophoresis for nonpregnant (NPC) and pregnant controls (PC) and patients with preeclampsia (PE1, PE2, PE3_1_, and PE3_2_ groups; case 18; and case 27).

Groups	HAS (%)	α_1_ (%)	α_2_ (%)	β_1_ (%)	β_2_ (%)	γ (%)
NPC	62.9 ± 1.8	3.8 ± 0.2	8.3 ± 1.2	6.1 ± 0.6	4.5 ± 1.2	14.5 ± 1.1
PC	49.0 ± 2.5 *	7.5 ± 1.4 *	15.2 ± 1.7 *	8.7 ± 1.1 *	8.6 ± 1.3 *	10.9 ± 1.7 *
PE1	49.2 ± 4.0	6.7 ± 1.5	14.8 ± 2.1	9.8 ± 2.0	7.2 ± 1.4	12.3 ± 2.7
PE2	51.7 ± 3.1	8.2 ± 1.1	12.8 ± 2.2 **	8.6 ± 2.1	7.7 ± 0.9	10.7 ± 2.5
PE3_1_	50.3 ± 3.1	7.2 ± 0.9	15.7 ± 1.8	9.4 ± 1.4	7.7 ± 1.8	6.7 ± 1.6 **
PE3_2_	51.5 ± 2.4	7.75 ± 0.7	13.8 ± 1.9	8.5 ± 0.8	8.8 ± 1.7	10.2 ± 1.7
Case18 Case27	35.86 ** 51.34	9.59 ** 7.14	25.18 ** 15.69	8.21 11.39 **	8.15 7.72	13.01 ** 6.71 **

* Indicates statistically significant difference (*p* < 0.05) in the PC values compared with the NPC values. ** Indicates statistically significant difference (*p* < 0.05) in the PC values.

**Table 5:** Main calorimetric parameters (mean value ± SD) estimated from the calorimetric profiles of newly isolated blood plasma from healthy nonpregnant women (NPC) and NPC plasma treated with 25 mM and 100 mM of H_2_O_2_.

Samples	ΔH (J·g^−1^)	T_FM_ (°C)	c_P_^HSA^/c_P_^Igs^
NPC	17.4 ± 0.5	65.8 ± 0.6	1.69 ± 0.06
NPC + 25 mM H_2_O_2_	14.7 ± 0.3	67.7 ± 0.4	1.20 ± 0.05
NPC + 100 mM H_2_O_2_	13.5 ± 0.6	67.9 ± 0.7	0.91 ± 0.09

**Table 6:** Main calorimetric parameters (mean value ± SD) estimated from the calorimetric profiles of purified HSA in its free form and after interaction with 25 and 100 mM of H_2_O_2_.

Samples	T_m_ (°C)	ΔH (J·g^−1^)
HSA	62.0	9.8
HSA + 25 mM of H_2_O_2_	61.7	8.4
HSA + 100 mM of H_2_O_2_	61.4	4.1

## Data Availability

All the data is contained within the article.
